# Impact of rapid diagnostic tests for the diagnosis and treatment of malaria at a peripheral health facility in Western Uganda: an interrupted time series analysis

**DOI:** 10.1186/s12936-015-0725-0

**Published:** 2015-05-15

**Authors:** Ross M. Boyce, Anthony Muiru, Raquel Reyes, Moses Ntaro, Edgar Mulogo, Michael Matte, Mark J. Siedner

**Affiliations:** Department of Medicine, Massachusetts General Hospital, 55 Fruit Street, Boston, 02114 USA; Center for Global Health, Massachusetts General Hospital, 100 Cambridge Street #1540, Boston, 02114 USA; Department of Community Health, Mbarara University of Science & Technology, P.O. Box 1410, Mbarara, Uganda

**Keywords:** Malaria, Rapid diagnostic tests, Outcomes, Case management, Antibiotics

## Abstract

**Background:**

The World Health Organization recommends that all suspected malaria cases receive a parasitological diagnosis prior to treatment with artemisinin-based combination therapy. A recent meta-analysis of clinical trials evaluating RDTs for the management of patients with fever found substantial reductions in anti-malarial prescriptions when health workers adhered to treatment protocols based on test results. However few studies have reported on the impact of RDTs on health systems outside research settings.

**Methods:**

The study comprised a retrospective interrupted time series analysis, comparing rates of malaria diagnosis, treatment, and resource utilization before and after introduction of RDTs at a peripheral health facility in rural Western Uganda. The use of malaria diagnostic tests was graphically depicted throughout the study period and fit regression models to identify correlates of three outcomes of interest: (1) length of stay (2) the proportion of patients referred to a higher-level health facility, and (3) administration of antibiotics.

**Results:**

Over the course of the study period, 14,357 individuals underwent diagnostic testing for malaria with either a RDT (9,807) or microscopy (4,550). The proportion of patients with parasite-based diagnoses more than tripled to 34 % after the introduction of RDTs. RDTs largely replaced microscopy as the diagnostic method of choice. Compared to patients admitted during the pre-RDT period, patients admitted to the health centre with malaria in the post-RDT period had significantly reduced odds of being referred to another health centre (AOR = 0.49, *P* = 0.038), receiving antibiotics (AOR = 0.42, *P* < 0.001), and a significantly shorter mean length of stay (β = −0.32 days, 95 %CI −0.52 to −0.13).

**Conclusions:**

This study is one of the few to demonstrate significant improvement in clinical outcomes and process measures following the introduction of RDTs for the diagnosis of malaria at a rural health facility in Uganda. The results show a reduction in referrals and shorter mean inpatient LOS even as antibiotics were prescribed less frequently. This change greatly increased laboratory throughput and the resultant proportion of patients receiving a parasite-based diagnosis.

## Background

Since 2010, the World Health Organization (WHO) has recommended that all suspected malaria cases receive a parasitological diagnosis, either by microscopy or a rapid diagnostic test (RDT), prior to treatment with artemisinin-based combination therapy (ACT) [1]. Light microscopy is the traditional reference standard for the diagnosis of malaria [1]. However, the vast majority of malaria episodes occur in rural areas of sub-Saharan Africa, where microscopy is often unavailable and shortages of trained personnel further limit its use as a valid diagnostic method [2–10]. Immunochromatographic RDTs are low-cost, simple tools for the diagnosis of malaria. In contrast to microscopy, RDTs require minimal infrastructure, can by used by non-professional healthcare staff, and provide a timely result [11].

A recent Cochrane Review of clinical trials evaluating RDTs for the management of patients with fever found substantial reductions in anti-malarial prescriptions when health workers adhered to treatment protocols based on test results [12]. However, the included studies demonstrated significant heterogeneity in clinical outcomes, both across and within a variety of study settings. The authors concluded that while the introduction of RDTs did not clearly improve health outcomes, adherence to the test result did not seem to result in worse outcomes than presumptive treatment strategies.

Few studies have reported on the impact of RDTs on health systems outside research settings, leading some to suggest that the existing evidence is insufficient to abandon other diagnostic modalities [13]. The study team attempted to address this knowledge gap through a retrospective investigation of clinical data before and after implementation of routine RDT availability at a peripheral health centre in rural Uganda. The working hypothesis was that the implementation of RDTs would increase the proportion of patients with a parasite-based diagnosis of malaria compared to microscopy alone, leading to more accurate and timely clinical case management and improved resource stewardship.

## Methods

### Study site

The Bugoye Level III Health Centre (BHC), in the Kasese District of Western Uganda (0° 18′ North, 30° 5′ East) functions as the referral centre for the Bugoye sub-county, serving a rural population of approximately 50,000 residents. The health centre is staffed by clinical officers, nurses, midwives, and laboratory technicians from the Ugandan Ministry of Health. The health centre is composed of an outpatient clinic that attends to approximately 60 patients per day, a 20-bed inpatient ward and a 10-bed maternity ward. A limited formulary of intravenous fluids, antibiotics, and anti-malarials, including oral artemether-lumafantrine and intravenous quinine, is available. The health centre houses a small laboratory with staff trained to perform light microscopy, as well other basic tests, such as hemoglobin estimation and rapid-HIV tests. The climate in Bugoye permits stable, year-round malaria transmission, with test positivity rates that can reach upwards of 50 % (unpublished data). RDTs were first introduced at BHC in the summer of 2011, although monthly supplies were initially highly variable.

### Study design

The study was a retrospective interrupted time series, comparing rates of malaria diagnosis, treatment, and resource utilization before and after introduction of RDT.

The timeframe of September 2010 to April 2014 was sub-divided into three periods: (1) the period prior to the introduction of RDTs when microscopy was the only method of malaria diagnosis available (pre-RDT period), (2) the 20 months following the introduction of RDTs but when their availability remained inconsistent (transition period), and (3) the period after March 2013, when a regular supply of RDT tests was available (RDT period). The transition period was included to account for the time required to assure a stable supply of test equipment, adherence to diagnostic and treatment algorithms, and appropriate use of the RDT under field conditions [12].

Six months of high transmission intensity were selected in each period from which to abstract clinical and programmatic information. In order to maximize the sample size and limit the seasonal effect of malaria transmission on the analysis, the months of October through January and May through June when transmission is traditionally intense were included in the analysis. Information on monthly malaria diagnostic test use during the study period was collected from monthly Health Management Information System (HMIS) reports, which are government-mandated documents completed for all encounters at each public health facility.

In addition, the following data was abstracted from clinical records for all inpatients with a discharge diagnosis of malaria: age, gender, village of residence, co-morbid diagnoses, type and route of anti-malarials received, antibiotics received, length of stay, and discharge disposition. The analysis was limited to inpatients with a diagnosis of malaria because these records are generally more accurate and complete than similar outpatient registries at the study site.

### Statistical analysis

Data were entered into Microsoft Excel (Redmond, WA) and analysed with Stata 12.1 (College Station, TX). The use of malaria diagnostic tests was first graphically depicted throughout the study period in order assess for trends in RDT implementation. Patient characteristics were then summarized across the three study periods using the ANOVA test.

The statistical analysis was designed to identify correlates of three outcomes of interest: (1) length of hospital stay (LOS) in days for patients who improved and were subsequently discharged home, (2) proportion of patients referred to a higher-level health facility, and (3) administration of antibiotics in patients with a discharge diagnosis of malaria. The first two outcomes were chosen as surrogate indicators of effective therapy for malaria. Firsthand experience at the clinical site suggests that patients who demonstrate improvement are discharged home, and a shorter length of stay represents the appropriate administration of anti-malarial therapy. Conversely, those who do not improve with treatment are referred to the regional referral hospital. Antibiotic use was chosen as the third outcome of interest in order to investigate whether implementation of RDTs would decrease antibiotic use in patients with a diagnosis of malaria. Logistical regression models were utilized in the analysis of the two discrete outcomes (referral, antibiotic use), while a linear regression model was used for the LOS analysis.

The primary explanatory variable of interest was the time period (pre-RDT, transition, and post-RDT). Secondary variables included gender, age (divided as <5, 5–15, and >15 years old), access to care, presence of a comorbid diagnosis, and transmission season defined as high (May-Jul and Nov-Jan) or low (Feb-Apr and Aug-Oct). As a surrogate of access to care, a remote village was defined as one from which transportation costs typically exceed 5,000 Uganda Shillings (approximately $2USD), versus villages physically bordering the health centre, from where most patients walk to the facility and do not incur costs of transportation. Multivariable regression models including all variables that were significant in univariable models with a pre-specified *p*-value of <0.25 were then undertaken [14]. A resulting *p-*value of <0.05 was considered statistically significant in the final models.

### Ethics statement

Ethical approval of the study was provided by the institutional review boards of Partners Healthcare and the Mbarara University of Science and Technology. Informed consent was not required by the ethical review committees due to the programmatic nature of the data collection and retrospective review of data.

## Results

### RDT supply and utilization

**Fig. 1:**
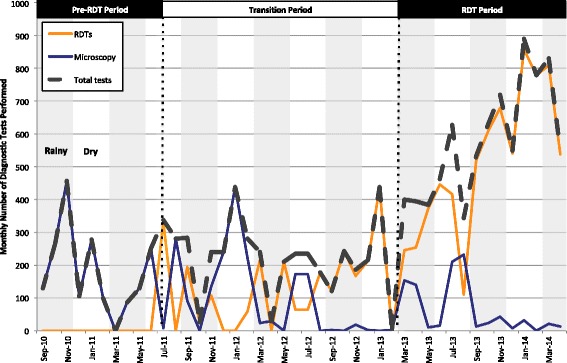
Trends in the use of malaria diagnostic tests (9/10–4/14)

Over the course of the study period, 14,357 individuals underwent diagnostic testing for malaria with either a RDT (9,807) or microscopy (4,550). Trends in the use of these tests during the observation period are shown in Fig. 1. There were no RDTs available in the pre-RDT period. The supply of RDTs was unstable in the transition period, with five months of complete stock-outs and four months of relative stock-outs. The majority (73 %) of RDTs were performed over the final 14 months of the study, when the supply became more consistent.

Figure 1 also highlights that the number of blood smears performed was inversely related to the number of RDTs performed. This is most clearly seen over the last six months of the transition period and throughout the RDT period, when RDTs were more readily available, and as a result, very few smears were performed.

While nearly 3,000 RDTs were performed at the health centre during the transition period, the proportion of patients with a diagnosis of malaria who received a parasite-based diagnosis increased only slightly, from 7.5 % to 9.4 %. The average number of total diagnostic tests, including both RDTs and microscopy, increased from only 180 per month in the pre-RDT period to 223 per month in the transition period. In contrast, the proportion of patients with parasite-based diagnoses more than tripled to 34 % during the RDT period with the proportion in the latter months consistently greater than 40 %. Over the same time period, the total number of diagnostic tests exceeds 500 tests per month for the first time and generally remained above that level.

### Impact of RDT implementation on clinical care

Over the 18 months of analysis, with six high transmission months from each period, a total of 1,106 patients were admitted to the ward and discharged with a diagnosis of malaria. Patient characteristics are summarized in Table 1.

**Table 1:** Characteristics of inpatients with a diagnosis of malaria in defined study periods

	Pre-RDT	Transition	Post-RDT	*P*-value
Patients (n, % of admissions)	300 (80.4)	282 (72.7)	524 (69.3)	0.33
Male Patients (n, %)	125 (42.2)	119 (42.5)	231 (44.3)	0.8
Age (median, IQR)	4 (1.25, 16)	9 (2.1, 21)	7 (3, 18)	0.27
<5 years	157 (52.3)	111 (39.36)	216 (41.2)	**0.003**
5–15 years	57 (19.0)	57 (20.2)	148 (28.2)	
>15 years	86 (28.67)	114 (40.43)	160 (30.5)	
Village (n, %)				
Bordering Villages	76 (26.6)	90 (33.1)	167 (33.2)	0.12
Remote Villages	46 (16.0)	39 (14.6)	38 (7.9)	**<0.001**
Comorbid diagnosis (n, %)				
Any comorbidity	150 (50.0)	153 (54.3)	144 (27.5)	**<0.001**
RTI/PNA	104 (34.7)	116 (41.1)	94 (17.9)	**<0.001**
Diarrhea	21 (7.0)	12 (4.3)	19 (3.6)	0.08
Anti-malarial (n, %)				
Intravenous	263 (88.7)	260 (92.2)	486 (93.1)	**0.024**
Oral only	35 (11.7)	19 (86.7)	33 (6.3)	**0.012**
Antibiotics	225 (75.0)	216 (76.6)	279 (53.6)	**<0.001**
LOS (mean, SD)	2.8 (1.3)	2.5 (1.2)	2.4 (1.0)	**<0.001**
Referred (n, %)	21 (8.0 %)	12 (4.5 %)	16 (4.2 %)	0.086

The proportion of patients < five years of age decreased significantly from 52.3 to 41.2 % of the total patient population, whereas there was a corresponding increase in patients between the ages of five and 15 (p = 0.003). The most common comorbidities among admitted patients were respiratory tract infections and/or pneumonia (28.4 %), diarrhoea and/or gastroenteritis (4.7 %), and HIV (1.5 %). The proportion of admitted patients with a documented comorbid diagnosis declined significantly (p < 0.001) in the RDT period with most of the decline attributable to a decrease in respiratory illnesses.

The majority (65.3 %) of patients admitted with a diagnosis of malaria received antibiotics, but the proportion decreased during the observation period, from 75.0 to 53.6 % from the pre- to post-RDT periods (p < 0.001). Similarly, the mean LOS decreased from 2.8 days (SD = 1.3) to 2.4 days (SD = 1.0, p < 0.001) and the proportion of patients referred to another health centre decreased from 7.4 to 4.2 % p = 0.086). These associations persisted in multivariable regression models (Tables 2, 3 and 4). Compared to patients admitted during the pre-RDT period, patients admitted to the health centre with malaria in the post-RDT period had significantly reduced odds of being referred to another health centre (AOR = 0.49, p = 0.038), receiving antibiotics (AOR = 0.42, p < 0.001), and a significantly shorter mean length of stay (β = −0.32, 95 %CI −0.52 to −0.13, p = 0.001), corresponding to a shorter mean hospital duration of stay by 0.32 days (approximately 8 hours) in the post-RDT period.

**Table 2:** Correlates of antibiotic administration for patients with a diagnosis of malaria

	Univariable model			Multivariable model*		
	OR	95 % CI	*P*-value	AOR	95 % CI	*P*-value
Male sex	1.24	0.97–1.6	0.09	1.24	0.94–1.63	0.12
Age						
>15	REF			REF		
5–15	0.98	0.71–1.35	0.88	-	-	-
<5	1.82	1.36–2.44	<0.001	1.97	1.44–2.70	**<0.001**
Remote	2.05	1.31–3.21	0.002	1.90	1.19–3.05	**0.007**
Referred	0.78	0.43–1.42	0.42	-	-	-
Period						
Pre-RDT	REF			REF		
Transition	1.09	0.75–1.60	0.65	-	-	-
RDT	0.38	0.28–0.53	<0.001	0.42	0.30–0.59	**<0.001**

*RDT* rapid diagnostic test, *OR* Odds ratio; *Explanatory variables with a *P*-value of <0.25 in the univariable model were included in the multivariable model

**Table 3:** Correlates of length of hospital stay (in days) for patients admitted with a diagnosis of malaria

	Univariable model			Multivariable model*		
	β	95 % CI	*P*-value	β	95 % CI	*P*-value
Male sex	0.05	−0.11–0.21	0.54	-	-	-
Age						
>15	REF			REF		
5–15	−0.17	−0.38–0.44	0.12	−0.12	−0.34–0.10	0.29
<5	0.01	−0.17–0.19	0.91	-	-	-
Remote	0.36	0.10–0.61	**0.006**	0.27	0.02–0.53	**0.033**
Antibiotics	0.23	0.07–0.40	**0.006**	0.17	−0.01–0.35	0.058
Period						
Pre-RDT	REF			REF		
Transition	−0.26	−0.46 to −0.06	**0.011**	−0.26	−0.46 to −0.05	**0.015**
RDT	−0.38	−0.56 to −0.19	**<0.001**	−0.32	−0.52 to −0.13	**0.001**

*RDT* rapid diagnostic test, *OR* Odds ratio; *Explanatory variables with a P-value of <0.25 in the univariable model were included in the multivariable model

**Table 4:** Correlates of referral for patients admitted with a diagnosis of malaria

	Univariable model			Multivariable model*		
	OR	95 % CI	*P*-value	AOR	95 % CI	*P*-value
Male sex	1.52	0.85–2.71	0.15	1.56	0.88–2.29	0.13
Age						
>15	REF			-	-	-
5–15	1.53	0.72–3.30	0.27	-	-	-
<5	1.13	0.57–2.26	0.73	-	-	-
Remote	1.27	0.55–2.92	0.57	-	-	-
Antibiotic	0.78	0.43–1.42	0.42	-	-	**-**
Period						
Pre-RDT	REF			REF		
Transition	0.55	0.26–1.13	0.11	0.54	0.26–1.12	0.099
RDT	0.51	0.26–0.99	0.046	0.49	0.25–0.96	**0.038**

*Explanatory variables with a P-value of <0.25 in the univariable model were included in the multivariable model

## Discussion

These results demonstrate significant decreases in inpatient LOS, referrals to higher-level facilities, and antibiotic administration among patients admitted to a rural health facility in Western Uganda with a diagnosis of malaria following the implementation of RDTs. Taken together, these findings suggest an improvement in clinical outcomes and health system resource allocation. This study, while retrospective, is among the first to detect a significant improvement in clinical outcomes attributable to the introduction of RDTs.

Notably, it was not until the RDT period, when the supply of diagnostic tests was more reliable, that a statistically significant decreases in the rates of antibiotic administration and referral was observed. This relationship further supports the hypothesis that the implementation of RDTs can improve the effectiveness of care delivery. The trend also suggests that much of the observed effect of RDTs may not be attributable to better test performance, but rather to the increase in laboratory capacity. These results suggest that a change to RDTs as the primary method of malaria diagnosis resulted in two- to three-fold increase in laboratory throughput, with a similar increase in the proportion of patients with a parasite-based diagnosis.

As the proportion of patients with a parasitologically confirmed diagnoses increased, there was a corresponding decrease in presumptive diagnosis and treatment, often referred to as “clinical malaria.” Health workers at the study site reported that they felt more certain about the diagnosis in patients with positive RDTs and thus did not list alternate etiologies, such as co-morbid respiratory tract infections or gastroenteritis, symptoms of which can be common with malaria infections [15, 16]. Health workers also commented that with greater confidence in the diagnosis, they were less likely to prescribe empiric antibiotics. The above findings reflect this sentiment with similar decrease in both co-morbid diagnoses and antibiotic prescriptions over the study period.

While these findings are intriguing, the near abandonment of microscopy merits further assessment. A shift to RDTs as the primary means of malaria diagnosis, as seen in other studies [17], raises concerns about the maintenance of critical laboratory proficiency. Microscopy offers the advantages of malaria species identification and estimates of parasite density. The poor quality of routine microscopy at many peripheral health centres likely over-estimates this limitation [2–4, 18] and preliminary work suggests that RDTs may be able to provide some quantitative estimate of parasite density [19–21]. Additionally, in areas of *P. falciparum* predominance, such as Uganda [22], the clinical utility of species identification is limited.

The study does have a number of methodological limitations. First, the study was ecological in design and thus assumes that the changes observed over the three defined periods were primarily attributable to the introduction of RDTs rather than other unmeasured factors. One will note there was a large increase in the number of admitted patients in the RDT period. This may be due to severe flooding that cut off nearby local villages from other health centres. It is reassuring, however, that the proportion of patients admitted with a diagnosis of malaria was not significantly different (80 % *vs.* 69 %, p = 0.33). Moreover, the consistent effect of RDTs, similar to a dose–response relationship, seen across the three study periods also partially mitigates this concern.

Second, HMIS reports and inpatient registries served as a primary data source for the analysis. These records are often incomplete and/or of variable accuracy, and thus could bias the above estimates. However, there is no reason to believe that the completeness or accuracy of the records changed over time, which would cause a differential effect by study period. The study team attempted to minimize this source of bias in three main ways. First, the team reviewed all data during the period of analysis and found no missing period or data points in the HMIS reports, and no evidence of suspicious entries, such as multiple months with identical values. Second, every effort was made to validate these results, including cross-referencing different sources such as laboratory records. Lastly, and most important, clinical information about individual patient diagnoses, treatment, and disposition was abstracted directly from the inpatient registrars. In the author’s experience in Bugoye, these records are generally of higher quality, which was one of the primary reasons for using this dataset as the basis for the analysis. For the three specified outcomes of interest, the individual data was available from clinical registries for greater than 80 % of admitted patients.

Third, while the study suggests improved clinical outcomes and a reduction in antibiotic administration in admitted patients with a diagnosis of malaria, it did not examine the impact on patients without a documented diagnosis of malaria or those with negative RDT results.

Despite these limitations, this natural experiment provides a unique look at the relationship between supply, capacity, diagnoses, and clinical outcomes. In fact, one of the strengths of the study is that there was no additional study-related training, monitoring, or funding. This characteristic enables consideration of these data as a real-world assessment of the implementation of RDTs, and augments the generalizability to other non-experimental clinical sites in rural Africa.

## Conclusions

This study is one of the few to demonstrate significant improvement in clinical outcomes and process measures following the introduction of RDTs for the diagnosis of malaria at a rural health facility in Uganda. The results demonstrate a significant reduction in referrals and shorter mean inpatient LOS even as antibiotics were prescribed less frequently. RDTs essentially replaced microscopy as the diagnostic method of choice. This change greatly increased laboratory throughput and the resultant proportion of patients receiving a parasite-based diagnosis. Future studies should evaluate the wider implications of a RDT-based strategy before further definitive policy change can be recommended.
